# Associated factors of the quality of therapeutic alliance in people with severe mental illness: A systematic review

**DOI:** 10.1192/j.eurpsy.2025.873

**Published:** 2025-08-26

**Authors:** M. Tetzlaff, J. Bruins, S. Castelein

**Affiliations:** 1 Research, Lenits Psychiatric Institute; 2 Faculty of Behavioral and Social Science, University of Groningen; 3 Rob Giel Research center, University Medical Center Groningen, University Center for Psychiatry, Groningen, Netherlands

## Abstract

**Introduction:**

Given the high rates of disengagement of psychological and/or psychopharmacological treatment in individuals with severe mental illness (SMI), building a strong therapeutic alliance (TA) in treatment is crucial. Despite the awareness of a favorable role of the TA in mental health care for people with SMI, there is a paucity of research that contributes to the formulation of concrete guidelines for establishing a strong TA.

**Objectives:**

This review aims to systematically synthesize existing literature of associated factors of the TA across six domains: client, mental health professionals (MHPs), clinical, social, care, and other. These include the views of clients with SMI, MHP, and independent raters of the TA.

**Methods:**

Parallel literature searches in PsycInfo, Medline, and PubMed between 2000-2022 identified 2699 possible articles, of which n=53 met inclusion criteria.

**Results:**

Associated factors of better client-rated TA were: high insight, secure attachment, higher outcome expectancy at baseline, specific personality traits, less internalized stigma, more therapists’ empathy and frequent use of supportive techniques by MHP. MHP-rated and/or independent observer-rated TA was significantly related to: more insight, sex of client (female), MHP without anxious attachment, and less severe symptomatology of client.

**Conclusions:**

Clinical symptom severity only affected TA when rated by MHP, but not when rated by clients. Attachment style affects the TA bidirectionally: clients’ secure attachment to the MHPs may help modify maladaptive attachment patterns, and anxious/insecure attachment style from either client or MHP affects the TA negatively. Furthermore, having an early positive click with the client builds the foundation for a later stable and supportive relationship, making the client more likely to continue perceiving the alliance as positively as treatment progresses. It is therefore crucial to provide a warm and supporting environment from the start of treatment, where clients have the opportunity to overcome perceived (self-)stigma and develop a positive mindset towards outcome expectations. Focusing on supportive techniques like providing feedback or shared agenda setting instead on the clients clinical symptomatology solely might result in a more favorable perception of the TA. Notably, current TA measurements assume a one-on-one relationship between clients and MHP, while nowadays multiple MHPs are involved. We recommend re-evaluating the assessment of TA within SMI care.

**Disclosure of Interest:**

None Declared

